# Clinical genome sequencing in neurodegenerative diseases–outcome in the first 500 patients

**DOI:** 10.1093/hmg/ddag072

**Published:** 2026-08-06

**Authors:** Emma Ehn, Håkan Thonberg, Inger Nennesmo, Anna Lindstrand, Malin Kvarnung, Caroline Graff

**Affiliations:** Division for Neurogeriatrics, Centre for Alzheimer Research, Department of Neurobiology, Care Sciences and Society, Karolinska Institutet, H1 Neurobiology, Care Sciences and Society, H1 Neurogeriatrics, 171 77 Stockholm, Sweden; Karolinska University Hospital Solna, Unit for Hereditary Dementias, Karolinska vägen 37A, 171 76 Stockholm, Sweden; Karolinska University Hospital Solna, Department of Clinical Genetics and Genomics, Karolinska Universitetslaboratoriet, Gävlegatan 68, 171 76 Stockholm, Sweden; Karolinska University Hospital Solna, Department of Clinical Genetics and Genomics, Karolinska Universitetslaboratoriet, Gävlegatan 68, 171 76 Stockholm, Sweden; Department of Molecular Medicine and Surgery, Karolinska Institutet, Karolinska University Hospital, Solna (L1:00), 171 76 Stockholm, Sweden; Department of Oncology-Pathology, Karolinska Institutet, OnkPat, 171 77 Solna, Sweden; Department of Pathology and Cancer Diagnostics, Karolinska University Hospital Solna, Klinisk Patologi/Cancerdiagnostik, Karolinska Universitetssjukhuset, Solna, 171 76 Solna, Sweden; Karolinska University Hospital Solna, Department of Clinical Genetics and Genomics, Karolinska Universitetslaboratoriet, Gävlegatan 68, 171 76 Stockholm, Sweden; Department of Molecular Medicine and Surgery, Karolinska Institutet, Karolinska University Hospital, Solna (L1:00), 171 76 Stockholm, Sweden; Karolinska University Hospital Solna, Department of Clinical Genetics and Genomics, Karolinska Universitetslaboratoriet, Gävlegatan 68, 171 76 Stockholm, Sweden; Department of Molecular Medicine and Surgery, Karolinska Institutet, Karolinska University Hospital, Solna (L1:00), 171 76 Stockholm, Sweden; Division for Neurogeriatrics, Centre for Alzheimer Research, Department of Neurobiology, Care Sciences and Society, Karolinska Institutet, H1 Neurobiology, Care Sciences and Society, H1 Neurogeriatrics, 171 77 Stockholm, Sweden; Karolinska University Hospital Solna, Unit for Hereditary Dementias, Karolinska vägen 37A, 171 76 Stockholm, Sweden

**Keywords:** neurodegeneration, Neurogenetics, rare variants, whole genome sequencing

## Abstract

Neurodegenerative diseases (NDDs) are clinically and genetically heterogeneous, requiring neuropathology or molecular testing for a definitive diagnosis. Clinical whole genome sequencing (WGS) enables comprehensive variant calling across flexible gene lists that can be tailored to the clinical presentation. By allowing simultaneous detection of single-nucleotide variants, copy-number variants, structural variants, and repeat expansions, WGS has the potential to improve diagnostic yield, facilitate genetic counseling and support clinical trial inclusion. This study assesses the diagnostic performance of WGS in individuals with NDD. WGS in 500 individuals representing a wide spectrum of NDDs identified a disease-causing variant in 61 cases, resulting in a diagnostic yield of 12%. These variants were found in 16 different genes, with *C9orf72* being the most prevalent. Repeat expansions represented the largest variant class, accounting for 35 of 61 LP/P cases (57%); most of which were *C9orf72* expansions (31/35). In the largest phenotype groups, frontotemporal dementia (FTD) had the highest diagnostic yield (19%) followed by amyotrophic lateral sclerosis (ALS, 13%), whereas an underlying monogenic cause was expectedly low in Alzheimer disease (AD, 4%). A positive family history was present in the majority (74%) of FTD, ALS, combined ALS-FTD and AD cases with an LP/P finding. Clinical WGS provides a clear diagnostic advantage in NDDs marked by substantial clinical and genetic overlap. WGS enables comprehensive variant detection and mapping of genotype–phenotype relationships across the disease continuum. In FTD and ALS, these results support universal access to genetic testing independent of age at onset or family history.

## Introduction

Neurodegenerative diseases (NDDs) encompass a heterogeneous group of disorders that affect the central nervous system with a progressive loss of neurons. NDDs include, but are not limited to, dementias (*e.g.* Alzheimer disease (AD) and frontotemporal dementia (FTD)), motor neuron diseases (MND) (*e.g.* amyotrophic laterals sclerosis (ALS)) and Parkinson’s disease (PD), which all have an adult onset and a progressive clinical course. Many NDDs have overlapping symptoms and share common biomarkers, which make clinical diagnostics challenging [1]. To obtain a definitive diagnosis, a postmortem neuropathological examination or molecular genetic testing is required. Despite intense research over decades, no curative treatment is yet available.

Genetic variants are central to the etiology of NDDs, either as monogenic causes or as risk factors in a multifactorial etiology [2]. Monogenic NDDs often represent rare Mendelian forms of common disorders, such as AD. A monogenic cause is more likely to be found in patients with early onset disease (<65 years) and/or a positive family history.

5–10% of all AD cases have an early onset (EOAD), and a monogenic cause in one of the three genes *APP*, *PSEN1* and *PSEN2* is found in about 5–10% of those cases [3].

FTD is the second most common early onset dementia with different clinical presentations including the behavioral variant (bvFTD), primary progressive aphasia (PPA), corticobasal syndrome or progressive supranuclear palsy. A positive family history is reported in about one third of FTD cases and in such cases, a monogenic cause is identified in up to 20% [4]. The most common disease-causing gene in FTD is *C9orf72,* followed by the genes *GRN* and *MAPT* [5].

Furthermore, FTD is on the same phenotypic continuum as ALS with up to 50% of people with ALS developing behavioral symptoms and 30% of individuals with FTD displaying motor symptoms [6]. This phenotypic overlap is supported by a genetic overlap with the *C9orf72* repeat-expansion underlying both diseases [7, 8]. Overall, 10% of ALS patients have a monogenic cause with a variant in one of the genes *C9orf72*, *SOD1*, *FUS* and *TARDBP* [9].

Due to etiological heterogeneity and the overlap in clinical manifestations across NDDs, short read whole genome sequencing (hereafter abbreviated as WGS) combined with phenotype-guided filtering of a broad disease gene list is an effective diagnostic strategy to establish a molecular diagnosis. WGS supports the detection of a wide spectrum of variant types—including SNVs, SVs, CNVs, and STRs—while certain complex, large-scale chromosomal rearrangements and some repeat expansions remain more challenging to resolve. In a clinical setting, WGS has successfully increased the number of genetically diagnosed cases in rare diseases [10–12].

In this descriptive, retrospective study we evaluate the outcome, diagnostic yield and clinical utility of WGS first in 500 individuals with NDDs.

## Results

### Patient cohort

The great majority (415) of the first 500 patients referred for mutation screening using WGS and an NDD gene list belonged to one of the phenotype groups; AD, FTD or ALS (Table 1).

**Table 1:** Phenotype groups and diagnostic yield in the total cohort. The number of identified likely pathogenic (LP) and pathogenic (P) findings for each group is summarized in the ‘LP/P findings’ column.

**Phenotype group**	**n**	**LP/P findings**	**Age, median (IQR)**	**Age range**	**Female (%)**
Azheimer Disease	149	6	66 (62–74)	51–91	70
Frontotemporal Dementia	136	25	73 (65–77)	45–93	46
Amyotrophic Lateral Sclerosis	130	17	64.5 (55–74)	20–84	42
Other	26	2	71 (59.5–78.5)	5–86	50
Parkinson Disease	21	2	57 (43–67)	30–83	57
Dementia Not Otherwise Specified	9	1	67 (65–77)	48–80	78
Combined Amyotrophic Lateral Sclerosis and Frontotemporal Dementia	8	2	64 (58–76.2)	42–78	50
Dementia with Lewy Bodies	6	0	71.5 (64.5–80)	62–83	17
Motor Neuron Disease Not Otherwise Specified	5	1	56 (54–78)	17–79	20
Ataxia	3	1	35 (31.5–56.5)	28–78	100
Primary Familial Brain Calcification	3	2	62 (62–62.5)	62–63	100
Cerebra Autosomal Dominant Arteriopathy with Subcortical Infarction and Leukoencephalopathy	2	1	77 (77–77)	77–77	100
Myeloneuropathy	1	1	57 (57–57)	57–57	0
Spastic paraparesis	1	0	24 (24–24)	24–24	100

The age of the patients varied from 5–93 years with a mean age of 66.4 years and there was a minor overrepresentation of females (53%). Only two children were included (one 5-year-old with suspected juvenile Huntington disease (phenotype group Neurodegeneration NOS) and one 17-year-old with MND NOS) and genetic screening was negative in both. Female representation was highest in the AD group (70%), compared with the ALS (42%) and FTD (46%) groups, see Table 1. Sex distribution differed significantly between AD and both FTD (p = 0.6 × 10^−4^) and ALS (p = 0.4 × 10^−5^), whereas no difference was observed between ALS and FTD (p = 0.6; Pearson’s χ^2^ test with Yates’ correction).

### Genetic results

#### Diagnostic yield

The diagnostic yield in the cohort was 12% (61/500), meaning that a likely pathogenic (LP) or pathogenic (P) variant was identified in 61 referrals providing a molecular diagnosis (Table 2). In addition, 8 variants of uncertain significance (VUS) and 7 variants in risk-modifying genes (*SORL1*, *GBA*) were detected (see Supplementary Table 1).

**Table 2:** Details of all likely pathogenic (LP) and pathogenic (P) variants. ALS = amyotrophic lateral sclerosis, AD = Alzheimer disease, FTD = frontotemporal dementia, MND = motor neuron disease, PD = Parkinson disease. NA = not applicable, n = number of individuals carrying the variant.

**Gene**	**n**	**Phenotype group**	**ACMG class**	**Variant details**	**Transcript**	**Protein prediction**	**Zygosity**
*AR*	1	Dementia NOS	P	41 repeats		expansion	Hemizygous
*ATP1A3*	1	PD	LP	c.2934G > A	NM_152296	p.Trp978*	Heterozygous
*C9orf72*	2	AD	P	>145		expansion	Heterozygous
*C9orf72*	13	ALS	P	>145		expansion	Heterozygous
*C9orf72*	2	Combined ALS and FTD	P	>145		expansion	Heterozygous
*C9orf72*	14	FTD	P	>145		expansion	Heterozygous
*DCTN1*	1	ALS	LP	c.279G > C	NM_004082	p.Gln93His	Heterozygous
*GRN*	1	FTD	P	c.1246dup	NM_002087.3	p.Cys416Leufs*30	Heterozygous
*GRN*	1	FTD	P	c.138 + 1_138 + 3del	NM_002087.3	splice site	Heterozygous
*GRN*	1	FTD	P	c.380_381del	NM_002087.3	p.Pro127Argfs*2	Heterozygous
*GRN*	1	FTD	LP	c.-9A > G	NM_002087.4	splice site	Heterozygous
*GRN*	1	FTD	LP	c.1414-2A > G	NM_002087.4	splice site	Heterozygous
*HTT*	1	FTD	P	41 CAG		expansion	Heterozygous
*NOTCH3*	1	CADASIL	P	c.1672C > T	NM_000435	p.Arg558Cys	Heterozygous
*PRKN*	1	PD	P	del exon 2 (154 kb), del exon 3 (54 kb)	NM_04562.3	deletion	Compound heterozygot
*PSEN1*	1	AD	LP	c.1270C > T	NM_000021	p.Leu424Phe	Heterozygous
*PSEN1*	2	AD	LP	c.392A > T	NM_000021	p.His131Leu	Heterozygous
*PSEN1*	1	AD	LP	c.616G > C	NM_000021	p.Gly206Arg	Heterozygous
*RFC1*	2	Other	P	400–2000 AAGGG		expansion	Biallelic
*SLC20A2*	1	PFBC	P	c.1802C > T	NM_001257180	p.Pro601Leu	Heterozygous
*SLC20A2*	1	PFBC	LP	c.1723G > A	NM_001257180.2	p.Glu575Lys	Heterozygous
*SOD1*	1	ALS	P	c.341 T > C	NM_000454	p.Ile114Thr	Heterozygous
*SOD1*	1	MND NOS	P	c.272A > C	NM_000454	p.Asp91Ala	Homozygous
*SOD1*	1	Myeloneuropathy	P	c.435G > C	NM_000454.4	p.Leu145Phe	Heterozygous
*TBK1*	1	FTD	P	c.1340 + 1G > A	NM_013254	splice site	Heterozygous
*TBK1*	1	FTD	LP	c.2086G > A	NM_013254	p.Glu696Lys	Heterozygous
*TBK1*	1	FTD	LP	c.557dup	NM_013254	p.Arg187Glufs*17	Heterozygous
*VAPB*	1	ALS	LP	c.166C > T	NM_001195677	p.Pro56Ser	Heterozygous
*VCP*	1	ALS	LP	c.273C > G	NM_007126	p.Asn91Lys	Heterozygous
*VCP*	1	FTD	LP	c.273C > A	NM_007126	p.Asn91Lys	Heterozygous
*VCP*	1	FTD	LP	c.278G > A	NM_007126.5	p.Arg93His	Heterozygous
*WDR45*	1	Ataxia	LP	c.992_1023del	NM_007075.4	p.Thr331Lysfs*12	Heterozygous

There was a significant difference in mean age at screening between patients with an LP/P variant (63.4 ± 11.2 years), and patients with no finding (66.9 ± 12.7 years) (p = 0.02 with Welch Two Sample *t-*test). No significant difference was observed in female/male distribution among individuals with an LP/P finding (number of females = 30, number of males = 31) and with no finding (number of females =231, number of males = 200), (p = 0.6 using Pearson’s Chi-squared test with Yates’ continuity correction). See Table 3.

**Table 3:** Age, sex demographics and outcome of the genetic screening in the overall cohort. VUS = variant of uncertain significance.

	**No finding**	**Pathogenic**	**Likely pathogenic**	**VUS**	**Overall**
	(N = 431)	(N = 45)	(N = 16)	(N = 8)	(N = 500)
**Sex**					
F	231 (54%)	20 (44%)	10 (63%)	6 (75%)	267 (53%)
M	200 (46%)	25 (56%)	6 (37%)	2 (25%)	233 (47%)
**Age**					
Mean (SD)	66.9 (12.7)	63.7 (10.9)	62.4 (12.2)	66.0 (6.23)	66.4 (12.5)
Median [Min, Max]	68.0 [5.0, 93.0]	65.0 [40.0, 79.0]	66.0 [35.0, 77.0]	66.0 [55.0, 74.0]	68.0 [5.0, 93.0]

Looking at diagnostic yield in the phenotype subgroups showed that it was 18% (25/136) in the FTD group, 13% (17/130) in the ALS group and only 4% (6/149) in the AD group. Among individuals with a combined ALS-FTD phenotype, two individuals had a *C9orf72* repeat expansion (2/8 = 25%) while six had no finding.

#### Disease genes (genes with LP/P variants)

A total of 16 different genes were implicated in the 61 patients where a molecular diagnosis was established. Most of these genes were each observed in only one patient. However, seven genes were recurrently identified in more than one patient (*C9orf72* n = 31, *GRN* n = 5, *SOD1* n = 3, *VCP* n = 3, *TBK1* n = 3, *PSEN1* n = 4, *SLC20A2* n = 2), see Table 2.

In some cases, genetic variants identified in genes such as *VCP*, and *C9orf72* were unexpected, as the observed phenotypes did not correspond to the classically described gene-associated presentations. LP variants in *VCP* were observed in patients with ALS as well as FTD and pathogenic repeat expansions in *C9orf72* were identified in individuals with ALS, AD, FTD and combined ALS-FTD. The distribution of *C9orf72* repeat expansions was 10% for both ALS (13/130) and FTD (14/136). In addition, two individuals with combined ALS-FTD (2/8) as well as two individuals clinically diagnosed with AD (2/149) (described as ‘atypical AD’ in medical records) had pathogenic repeat expansions in *C9orf72*.

Looking at the disease genes in the four largest phenotype groups (FTD, ALS, AD and combined ALS-FTD), again *C9orf72* was the most common gene in all groups except in the AD group in which *PSEN1* dominated and *C9orf72* was the second most common (Fig. 1). *C9orf72* carriers with FTD were significantly older at referral (68.9 ± 5.5 years) than those with ALS (56.8 ± 11.8 years) (*p* = 0.004 with Welch two-sample *t-*test).

**Figure 1:**
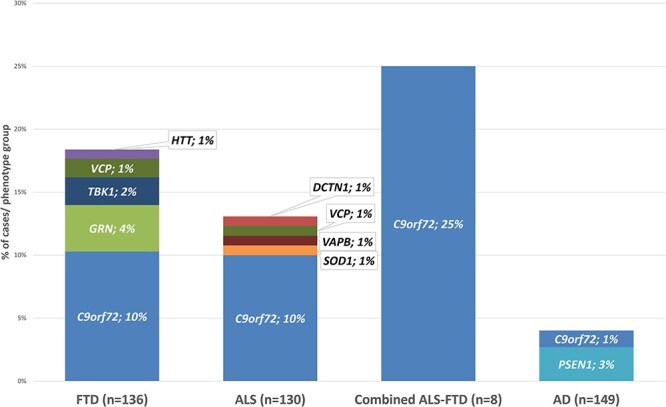
Distribution of genes with likely pathogenic (LP) or pathogenic (P) variants in the phenotype groups frontotemporal dementia (FTD), amyotrophic lateral sclerosis (ALS), combined ALS-FTD and Alzheimer disease (AD). Percentages shown in the bars indicate the proportion of individuals carrying variants in each gene relative to the total number of individuals within the respective phenotype group.

Postmortem neuropathology examination was available for 13 of 69 LP/P/VUS carriers. The neuropathology findings were concordant with the genetic findings across all cases. See Supplementary Table 1.

#### Variant spectrum

Repeat expansions were the most prevalent variant type, accounting for 57% (35/61) of the diagnostic cases and were detected across four genes (*AR*, *C9orf72*, *HTT*, *RFC1*). Missense (n = 16), frame shift (n = 4), splice site (n = 4) and nonsense (n = 1) alterations altogether accounted for 41% (25/61) of the findings. SVs were only detected in one individual with PD who carried a compound heterozygous deletion in *PRKN* (1/64). Except for repeat expansions in *C9orf72*, most variants were unique and identified in only one individual. However, the variant c.392A > T in *PSEN1* was identified in two individuals who were siblings. A great majority of the individuals (n = 55) carried heterozygous variants consistent with autosomal dominant diseases, four individuals carried bi-allelic variants, while the remaining two individuals carried variants on the X-chromosome (expansion in *AR* and an SNV in *WDR45*). See Table 2.

### Family history

Family history was assessed in individuals with largest phenotype groups (FTD, ALS, combined ALS-FTD or AD) who harbored an LP or P variant (n = 50) using the modified Goldman score (Table 4). A positive family history was present in 74% (at least one affected relative, score 1–3.5) and 42% demonstrated a strong family history consistent with autosomal dominant inheritance (score 1–2; Table 4). In contrast, 18% of these patients with a disease-causing variant had no reported first- or second-degree relatives with NDDs (score 4), and family history information was unavailable in 8% (score 4.5). Within phenotype-specific groups, a positive family history was most frequent in AD (100%) and FTD (80%), followed by ALS (53%). All six AD cases with a causative finding had a positive family history, of whom 83% met criteria for autosomal dominant inheritance (score 1). Both individuals with a combined ALS–FTD phenotype had a strong family history, and one exhibited a clear autosomal dominant pattern.

**Table 4:** Family history for the AD, ALS, FTD and ALS-FTD cases with a causative finding. Percentage may not sum to 100% due to rounding.

	**AD n = 6**	**ALS n = 17**	**Combined ALS and FTD n = 2**	**FTD n = 25**	**Overall n = 50**
**Modified Goldman score**					
1	5 (83%)	3 (18%)	1 (50%)	6 (24%)	15 (30%)
2	0 (0%)	2 (12%)	1 (50%)	3 (12%)	6 (12%)
3	1 (17%)	3 (18%)	0 (0%)	1 (4%)	5 (10%)
3.5	0 (0%)	1 (6%)	0 (0%)	10 (40%)	11 (22%)
4	0 (0%)	6 (35%)	0 (0%)	3 (12%)	9 (18%)
4.5	0 (0%)	2 (12%)	0 (0%)	2 (8%)	4 (8%)

## Discussion

In this study, we report the screening outcome in the first 500 individuals referred for WGS analysis using the NDD gene list. The overall diagnostic yield of 12% aligns with previous studies in combined dementia cohorts using WGS, exome sequencing, or targeted gene lists, despite differences in inclusion criteria and methods [13–15].

A key strength of this study is that the cohort reflects routine clinical practice, including patients referred from multiple regions and clinical specialties. Importantly, this cohort represents the phenotypic spectrum of the first 500 consecutive referrals for WGS with the NDD panel and does not mirror the broader phenotype distribution at the referring clinics. Among the phenotype groups, AD had the lowest yield (4%), again underscoring the rarity of deterministic variants in AD and confirming findings from earlier work [3, 16, 17]. In contrast, individuals with FTD and ALS showed markedly higher yields (19% and 13%, respectively), consistent with previous reports, although the genetic landscape differed in other cohorts [5, 18–23]. Across the cohort, the *C9orf72* repeat expansion was the most common pathogenic finding, dominating the ALS, FTD, and combined ALS–FTD subgroups, and is in line with findings from previous studies [5, 18, 19, 24].

A key strength of this study is the uniform application of a single WGS-based diagnostic pipeline, incorporating a comprehensive NDD gene panel, to all referred individuals irrespective of presenting phenotype. This approach enabled the identification of genetic etiologies beyond those suggested by the initial clinical diagnosis. Notably, we detected a pathogenic *HTT* expansion in a case referred with FTD as well as pathogenic *C9orf72* repeat expansions in two individuals referred with AD. Our findings suggest that *C9orf72* can present with various clinical presentations [17, 20]. This is supported by recent findings in population-based data from the UK Biobank showing that *C9orf72*-associated disease encompasses a broader and more heterogeneous clinical spectrum [25]. However, in our cohort we cannot exclude the possibility of clinical misclassification within the dementia spectrum as an explanation for the *C9orf72* findings in two AD patients. This further underscores the importance of a comprehensive, phenotype-independent genetic screening across the NDD spectrum.

Although SVs were rare in our cohort, their detection remains clinically important as SVs are known causes of NDD, including *APP* duplications and triplications in early-onset autosomal dominant AD [26–28]. Notably, no pathogenic or likely pathogenic variant was identified in *APP* or *PSEN2* during the study period. In contrast, several novel variants were detected in *PSEN1*. In the FTD phenotype group, *TBK1* and *VCP* contributed to multiple LP/P cases, while no pathogenic *MAP*T variants were detected, despite the gene’s well-established involvement in autosomal dominant FTD [20, 23]. On the other hand, absence of *MAPT* variants in genetic screening were previously reported in a Serbian dementia cohort including FTD patients [29].

Although individuals carrying LP/P variants were younger at referral and more often reported a positive family history—features consistent with monogenic disease [17, 23, 30]—our findings emphasize that these characteristics should not be used to restrict access to genetic testing in ALS and FTD. A substantial proportion of genetically determined cases occur in the absence of a known family history [17, 19, 31]. While previous studies have shown a lower diagnostic yield in sporadic compared with familial ALS, this does not preclude clinically relevant findings in apparently sporadic cases [24, 32].

WGS offers several advantages over exome-based methods, including detection of intronic repeat expansions, noncoding variants, and CNVs/SVs. As long-read sequencing becomes more integrated into clinical diagnostics, we anticipate further gains in diagnostic yield in NDDs, particularly through improved STR expansion-sizing, methylation analysis, and resolution of complex structural rearrangements [33]. Importantly, identifying a genetic cause enables a definitive diagnosis, reduces unnecessary clinical investigations, enables genetic counselling including reproductive alternatives, and—critically—provides access to targeted clinical trials. Recently approved *SOD1*-targeted therapies, along with ongoing clinical trials involving *GRN*, *C9orf72, FUS* as well as presymptomatic *SOD1* mutation carriers, further demonstrate how genetic diagnosis can shape access to therapeutic interventions—particularly in rapidly progressing disorders with a limited window for trial eligibility (see ClinicalTrials.gov; e.g. NCT06064890, NCT04747431, NCT04408625, NCT04768972, NCT04856982). These developments underscore the critical importance of identifying underlying genetic etiologies in ALS and FTD [34].

In light of the evolving therapeutic landscape, it is timely to reconsider the scope of clinical genetic reporting. Current practice prioritizes variants with well-established pathogenicity, often excluding disease-modifying genes. However, growing evidence supports the inclusion of relevant risk and modifier variants likely to influence disease pathogenesis, such as *GBA* in DLB/PD, *UNC13A* in *C9orf72*-associated disease, and *TMEM106B* in *GRN* mutation carriers [35, 36]. Reporting such findings may aid clinical trial stratification and provide a more comprehensive understanding of disease etiology, even when not suitable for predictive or prenatal use. A practical approach would be to present this information in a clearly delineated section, separate from definitively pathogenic variants.

In summary, given the high diagnostic yield observed in FTD and ALS, together with the substantial clinical implications of identifying a genetic etiology, we strongly advocate that all individuals with a clinical diagnosis of ALS and/or FTD—regardless of age at onset or family history—should be offered genetic screening. Such screening is preferably performed using WGS, incorporating comprehensive variant calling and phenotype-guided filtering based on a broad, clinically curated NDD gene list. Collectively, we hope that our findings can provide a basis for a broader discussion on genetic screening criteria and, importantly, on ensuring equitable access to genetic testing [37]. Although genetic findings are still missing in many cases, automated reanalysis of WGS data and future implementation of long-read sequencing hold significant promise for uncovering additional causes and improving diagnostic precision [38, 39].

Some limitations of the study should be acknowledged. First, the cohort is subject to ascertainment bias and therefore does not reflect all patients with NDD, as referrals were clinically prioritized. Accordingly, these data should not be considered appropriate for assessing the prevalence of variants across different phenotype groups. A non-prioritized prospective study including all patients diagnosed with NDDs would serve as a better ground to characterize the genetic landscape in these phenotype groups. Second, the retrospective design of the study limits the completeness and uniformity of the clinical data, as information on the referrals on phenotypic characteristics, age at onset, and family history varied considerably. Ethical regulations restricted access to comprehensive medical records, further contributing to incomplete clinical documentation. In addition, not all individuals were analyzed with the latest version of the gene panel, possibly missing some genetic variants. Third, family history was not systematically collected across the entire cohort and only assessed in individuals in the largest phenotype groups with a causative finding. Finally, we consistently had data on age at referral but not age at onset or age at diagnosis in all patients and therefore, any conclusions regarding age should be interpreted with caution.

## Materials and methods

### Study design and patient cohort

This study includes the first 500 patients with NDD referred for genetic screening with WGS and the NDD gene panel at Clinical Genetics and Genomics, Karolinska University Hospital, between January 13, 2021 until March 25, 2024. See Supplementary Table 2. The decision to refer patients for genetic testing was made by the referring clinicians, and the specific clinical criteria for referral were therefore non-uniform. Age at referral was calculated primarily as the difference between the calendar year of referral and the year of birth. For individuals whose genetic screening was performed on bio banked DNA, age at referral was instead calculated as the difference between the year of sampling and the year of birth. When the year of sampling was unavailable, the year of referral was used as a proxy. In rare cases where screening utilized DNA from a deceased individual and the year of sampling was unknown, age at referral was estimated as the difference between the year of death and the year of birth. Information on phenotype and family history (see below) was retrieved from the referrals and from medical records when available. Patients were grouped into 14 phenotype groups, see Table 1.

### Genome sequencing and bioinformatic analysis

Genomic DNA from peripheral blood was isolated in accordance with the clinical routine at Clinical Genetics and Genomics, Karolinska University Hospital as previously described [40]. Occasionally, DNA was extracted from saliva (n = 1) and brain tissue (n = 3), at an external facility and subsequently sent to our clinic for analysis.

WGS was done at Clinical Genomics, SciLifeLab, Solna, Sweden as described previously [10]. Briefly, a TruSeq DNA PCR-free library preparation was paired-end sequenced on NovaSeq X (30×) and assembly was made onto GRCh37, variant calling was done through the Mutation Identification Pipeline (MIP) https://github.com/Clinical-Genomics/MIP and delivered into the clinical ranking and visualization tool Scout (https://github.com/Clinical-Genomics/scout). Genome-wide sequencing data were analyzed using comprehensive variant calling and subsequently filtered through a clinically curated neurodegenerative disease gene panel. The panel was updated during the study period, increasing from 90 in version 4 to 136 genes in version 7, and including 15–17 repeat-expansion regions (see Supplementary Table 2). The gene list was based on OMIM phenotypes (https://www.omim.org/) and curation of Panel App Genomics England (Adult onset neurodegenerative disorder (Version 8.10), focusing on green genes with established disease association. Additionally, *SORL1* was included as a research candidate gene, and not as a strict clinical disease associated gene.

### Variant evaluation and classification

Public databases used for variant evaluation included (but were not restricted to) ClinVar, gnomAD (v2.1.1), OMIM and expert databases including https://www.alzforum.org (mutation database) and https://www.GENFI.org (FTD mutation list) for AD and FTD related genes respectively. In silico prediction tools included PolyPhen, SIFT, SPIDEX, REVEL and SpliceAI for variant severity and PHAST GERP and PhyloP for amino acid conservation. Variants were classified using the American College of Medical Genetics and Genomics (ACMG) criteria as either benign (B), likely benign (LB), variant of uncertain significance (VUS), likely pathogenic (LP) or pathogenic (P) [41].

### Validation of variants

All LP, P and reported VUS variants, including the challenging *GBA* region, were verified by secondary methods, either Sanger-sequencing or repeat-primed PCR and fragment length analysis. Repeat expansions in *C9orf72* and *HTT* were verified using commercial AmplideX® PCR/capillary electrophoresis (CE) kits (Asuragen, Inc.) where the threshold for pathogenicity is at > 145 repeats, following the manufacturer’s instructions. Verification of *RFC1* and *AR* expansions was performed in-house using PCR-based methods combined with capillary electrophoresis [42–44].

### Statistics and plots

Statistics and data processing were performed in RStudio version 2025.9.2.418. Version Posit team (2025), RStudio: Integrated Development Environment for R. Posit Software, PBC, Boston, MA. URL http://www.posit.co/. Sex differences were assessed using Pearson’s chi-square test with Yates’ correction, and median age was compared using Welch’s two-sample *t-*test. Statistical significance was defined as a p value < 0.05. Plots and tables were created in Microsoft Excel.

### Family history

Information on family history of NDDs was obtained from medical records for patients carrying P or LP variants within the AD, FTD, ALS, and combined ALS–FTD phenotype groups. A modified Goldman score (1–4.5), initially developed for FTD pedigrees, was used for family history evaluation [45–47]. To enable classification in the cohort, patients and relatives with NDDs including not only FTD but also AD, PD, DLB, ataxia and other NDDs were included and scored as 1 = Autosomal dominant inheritance with ≥3 affected relatives in 2 generations (one relative must be first degree relative (FDR) with the other two), 2 = ≥3 affected relatives but not meeting score 1, 3 = ≥1 affected first or second degree relative with onset ≤ 65 years, 3.5 = ≥1 affected first or second degree relative with onset > 65 years, 4 = no first- or second degree relative with NDD, 4.5 = unknown family history/information not available. When the age at onset of an affected FDR was unknown, a score of 3.5 was used.

### Neuropathology

For individuals carrying LP, P, or VUS variants, neuropathology examination reports were reviewed, with most information obtained through the Brain Bank at Karolinska Institutet research program and less frequently from medical records. See Supplementary Table 1.

## Ethics approval and consent to participate

The study was done according to the Helsinki Declaration and was approved by the Swedish Ethical Review Authority (ethics permit number 2012/222–31/3, addendum 2023–00178-02). The permit does not require written consent for genome sequencing. Approval of access to medical records was obtained from the neurology clinic under K2023–9920 (application) and Dnr K2023–9877 and K2023–9557 (decision).

## Supplementary Material

Supplementary_materials_ddag072

## Data Availability

The data supporting the findings of this study are available within the article and its supplementary material. Additional data may be made available by the corresponding author upon reasonable request and in accordance with applicable Swedish legislation.
