# Supplementary material for: Clinical genome sequencing in neurodegenerative diseases–outcome in the first 500 patients
Source: Hum Mol Genet. 2026 Aug 6;35(16):ddag072. doi: 10.1093/hmg/ddag072 (PMC13648039; doi:10.1093/hmg/ddag072)
Supplement: Supplementary file 1 [file supplementary_materials_ddag072.zip › Suppl_table_2_neurodegpanels_resubmission.pdf]

# Gene panel Neurodegeneration

Version 4.0 (2020-05-13)

90 genes, 15 repeat regions

## 90 genes

ALS2, ANG, APP, AR, ATN1, ATP13A2, ATP1A3, ATXN1, ATXN10, ATXN2, ATXN3, C19orf12, C9orf72, CHCHD10, CHMP2B, CSF1R, CYLD, DCTN1, DNAJC5, DNAJC6, DNMT1, EPM2A, ERBB4, FBXO7, FIG4, FTL, FUS, GBA, GCH1, GRN, HNRNPA1, HTT, ITM2B, JPH3, LRRK2, LYST, MAPT, MATR3, MME, NEF4 (PRPH), NEFH, NHLRC1, NIPA1, NOP56, NOTCH3, OPA3, OPTN, PANK2, PARK7, PFN1, PINK1, PLA2G6, PLD3, PPP2R2B, PRKN, PRKRA, PRNP, PSEN1, PSEN2, RAB39B, SETX, SIGMAR1, SLC30A10, SLC39A14, SLC52A2, SLC52A3, SLC6A3, SNCA, SOD1, SORL1, SPG11, SPR, SQSTM1, SYNJ1, TARDBP, TBK1, TBP, TH, TMEM106b, TREM2, TUBA4A, TUBB4A, TYROBP, UBE3A, UBQLN2, VAPB, VCP, WDR45, VPS13A, VPS35

## 15 repeat expansion regions

AR, ATN1, ATXN1, ATXN10, ATXN2, ATXN3, ATXN7, ATXN8OS, C9ORF72, CACNA1A, FXN, NOP56, PPP2R2B, RFC1, TBP

# Gene panel Neurodegeneration

Version 5.0.1 (2021-05-20)

123 genes, 17 repeat regions

## 123 genes

ABCD1, AFG3L2, ALS2, ANG, ANXA11, APP, AR (STR), ARSA, ATN1 (STR), ATP13A2, ATP1A3, ATP7B, ATXN1 (STR), ATXN10 (STR), ATXN2 (STR), ATXN3 (STR), ATXN7 (STR), ATXN8OS (STR), AUH, C19orf12, C9orf72 (STR), CACNA1A (STR), CACNA1G, CCNF, CHCHD10, CHCHD2, CHMP2B, CLCN2, CLN6, COASY, CP, CSF1R, CTSF, CYLD, CYP27A1, CYP7B1, DARS2, DCTN1, DNAJC5, DNAJC6, DNMT1, EIF2B1, EIF2B2, EIF2B3, EIF2B4, EIF2B5, ELOVL4, EPM2A, ERBB4, FBXO7, FIG4, FTL, FUS, FXN (STR), GBA, GCH1, GFAP, GRN, HEXA, HEXB, HNRNPA1, HTRA1, HTT (STR), ITM2B, JPH3 (STR), KCNC3, KCND3, KIAA1161, KIF5A, LRRK2, LYST, MAPT, MATR3, NHLRC1, NIPA1, NOP56 (STR), NOTCH3, NPC1, NPC2, OPTN, PANK2, PARK7, PDGFB, PDGFRB, PFN1, PINK1, PLA2G6, PLD3, PP2R2B (STR), PRKN, PRKRA, PRNP, PSEN1, PSEN2, RFC1 (STR), RNF216, SETX, SIGMAR1, SLC20A2, SLC30A10, SNCA, SOD1, SORL1, SPAST, SPG11, SQSTM1, SYNJ1, TARDBP, TBK1, TBP (STR), TMEM240, TREM2, TTC19, TUBA4A, TUBB4A, TYROBP, UBQLN2, VAPB, VCP, WDR45, VPS13A, VPS35, XPR1

## 17 repeat expansion regions

AR, ATN1, ATXN1, ATXN10, ATXN2, ATXN3, ATXN7, ATXN8OS, C9orf72, CACNA1A, FXN, HTT, JPH3, NOP56, PP2R2B, RFC1, TBP

## Gene panel Neurodegeneration

Version 6 (2021-09-08)

127 genes, 17 repeat regions

### 127 genes

ABCD1, AFG3L2, ALS2, ANG, ANXA11, APP, AR (STR), ARSA, ATN1 (STR), ATP13A2, ATP1A3, ATP7B, ATXN1 (STR), ATXN10 (STR), ATXN2 (STR), ATXN3 (STR), ATXN7 (STR), ATXN8OS (STR), AUH, C19orf12, C9orf72 (STR), CACNA1A (STR), CACNA1G, CCNF, CHCHD10, CHCHD2, CHMP2B, CLCN2, CLN6, COASY, CP, CSF1R, CTSF, CYLD, CYP27A1, CYP7B1, DARS2, DCTN1, DNAJC5, DNAJC6, DNMT1, EIF2B1, EIF2B2, EIF2B3, EIF2B4, EIF2B5, ELOVL4, EPM2A, ERBB4, FBXO7, FIG4, FTL, FUS, FXN (STR), GBA, GCH1, GFAP, GRN, HEXA, HEXB, HNRNPA1, HTRA1, HTT (STR), ITM2B, JPH3 (STR), KCNC3, KCND3, KIF5A, LRRK2, LYST, MAPT, MATR3, NHLRC1, MYORG, NIPA1, NOP56 (STR), NOTCH3, NPC1, NPC2, OPA3, OPTN, PANK2, PARK7, PDGFB, PDGFRB, PFN1, PINK1, PLA2G6, PLD3, PP2R2B (STR), PRKN, PRKRA, PRNP, PRPH, PSEN1, PSEN2, RAB39B, RFC1 (STR), RNF216, SETX, SIGMAR1, SLC20A2, SLC30A10, SNCA, SOD1, SORL1, SPAST, SPG11, SPR, SQSTM1, SYNJ1, TARDBP, TBK1, TBP (STR), TMEM240, TREM2, TTC19, TUBA4A, TUBB4A, TYROBP, UBQLN2, VAPB, VCP, WDR45, VPS13A, VPS35, XPR1

### 17 repeat expansion regions

AR, ATN1, ATXN1, ATXN10, ATXN2, ATXN3, ATXN7, ATXN8OS, C9orf72, CACNA1A, FXN, HTT, JPH3, NOP56, PP2R2B, RFC1, TBP

## Gene panel Neurodegeneration

Version 7 (2023-04-24)

136 genes, 17 repeat regions

### 136 genes

ABCD1, AFG3L2, ALS2, ANG, ANXA11, APP, AR (STR), ARSA, ATN1 (STR), ATP13A2, ATP1A3, ATP7B, ATXN1 (STR), ATXN10 (STR), ATXN2 (STR), ATXN3 (STR), ATXN7 (STR), ATXN8OS (STR), AUH, C19orf12, C9orf72 (STR), CACNA1A (STR), CACNA1G, CCNF, CHCHD10, CHCHD2, CHMP2B, CLCN2, CLN6, COASY, COL4A1, COL4A2, CP, CSF1R, CTSA, CTSF, CYLD, CYP27A1, CYP7B1, DARS2, DCTN1, DNAJC5, DNAJC6, DNMT1, EIF2B1, EIF2B2, EIF2B3, EIF2B4, EIF2B5, ELOVL4, EPM2A, ERBB4, FBXO7, FIG4, FTL, FUS, FXN (STR), GBA, GCH1, GFAP, GLA, GRN, GSN, HEXA, HEXB, HNRNPA1, HTRA1, HTT (STR), ITM2B, JPH3 (STR), KCNC3, KCND3, KIF5A, LAMB1, LRRK2, LYST, MAPT, MATR3, NHLRC1, MYORG, NIPA1, NOP56 (STR), NOTCH3, NPC1, NPC2, OPA3, OPTN, PANK2, PARK7, PDGFB, PDGFRB, PFN1, PINK1, PLA2G6, PLD3, PP2R2B (STR), PRKN, PRKRA, PRNP, PRPH, PSEN1, PSEN2, RAB39B, RFC1 (STR), RNF216, SETX, SIGMAR1, SLC20A2, SLC30A10, SNCA, SOD1, SORL1, SPAST, SPG11, SPR, STUB1, SQSTM1, SYNJ1, TARDBP, TBK1, TBP (STR), TMEM240, TREM2, TREX1, TTC19, TTR, TUBA4A, TUBB4A, TYROBP, UBQLN2, VAPB, VCP, WDR45, VPS13A, VPS35, XPR1

## 17 repeat expansion regions

AR, ATN1, ATXN1, ATXN10, ATXN2, ATXN3, ATXN7, ATXN8OS, C9orf72, CACNA1A, FXN, HTT, JPH3, NOP56, PP2R2B, RFC1, TBP
